# Effect of Surface Treatments Applied to 3D Printed Permanent Resins on Shear Bond Strength

**DOI:** 10.4317/jced.61884

**Published:** 2024-09-01

**Authors:** Bilge Ersöz, Numan Aydın, Bahadır Ezmek, Serpil Karaoğlanoğlu, İrem-Kübra Çal

**Affiliations:** 1 DDS, PhD. University of Health Sciences, Gulhane Faculty of Dentistry, Department of Restorative Dental Treatment, Ankara, Turkey; 2 DDS, PhD. University of Health Sciences, Gulhane Faculty of Dentistry, Department of Prosthodontics, Ankara, Turkey; 3 DDS. University of Health Sciences, Gulhane Faculty of Dentistry, Department of Restorative Dental Treatment, Ankara, Turkey

## Abstract

**Background:**

3D-printed permanent resins have recently been introduced to produce permanent restorations. The aim of this study is to evaluate the effect of surface treatment methods on the shear-bond strength (SBS) between 3D-printed permanent resins and adhesive cement.

**Material and Methods:**

In this study, samples were produced using digital light projection (DLP) and stereolithography (SLA) 3D printers with two permanent resins (Crowntec, Saremco and Permanent Crown, Formlabs) in accordance with manufacturer guidelines. The samples were separated into three groups: sandblasting, hydrofluoric acid and no surface treatment. The surface profile (Ra, Sa) of the samples was examined with a confocal microscope (Smartproof 5, Zeiss). Then, a self-adhesive resin cement was applied to the samples in a transparent mold (2.38 mm diameter) in accordance with ISO 29022:2013. A universal testing machine was used to perform SBS test. A stereomicroscope was used to analyze the different types of fractures. A two-way analysis of variance (ANOVA) test was used to assess the data (*p*<0.05).

**Results:**

The samples with sandblasting applied to the surface showed higher surface roughness values than the samples with hydrofluoric acid (*p*<0.001). 3D printer technology and surface treatment methods affected SBS values (*p*<0.001). Sandblasting groups higher SBS values were than in the hydrofluoric acid group (*p*<0.001). Sandblasting revealed cohesion fractures, which indicated a stronger bond, while hydrofluoric acid displayed adhesive and mix fractures.

**Conclusions:**

When sandblasting was applied to the surface of the samples prepared using permanent resins, higher adhesion was achieved with adhesive cement.

** Key words:**3D printing, Shear bond strength, Sandblasting, Stereolithography.

## Introduction

The dental industry has just started to adopt three-dimensional (3D) printers (1). Additive manufacturing technology 3D printing has the advantages of a shorter manufacturing time, reduced material consumption, and no waste material (2). Based on printing methodology, 3D printers are classified into seven groups: binder jetting, vat photopolymerization, material extrusion or fused deposition modeling, material jetting, powder bed fusion, sheet lamination, and direct energy deposition (3).

Vat photopolymerization includes stereolithography (SLA) and digital light projection (DLP). The main difference between these technologies is the light source. While SLA uses ultraviolet (UV) laser to polymerize point by point of the layer, (4) DLP uses high-power LED to polymerize the entire layer in two dimensions (x/y axes) at the same time (5). SLA technology has the advantages of high resolution, fine precision, and suitability for functional prototyping, while DLP technology has the advantages of high print resolution, fast production, and lower production costs (6).

3D printers in dentistry may create surgical guides, occlusal splints, definitive casts, full dentures, and temporary crowns and bridges (7). Ceramic was added to the resin printing medium to improve its esthetic, durable, and biocompatible qualities. This allowed for the printing of permanent crowns, bridges, and inlay-onlay restorations in three dimensions. It has been observed that 3D-printed permanent resins possess the necessary hardness and compressive and tensile strength for intraoral application (8). To polymerize unreacted monomers and guarantee uniform and full polymerization throughout all products, 3D printed goods must undergo a post-curing procedure (9).

The strength and durability of the bond between indirect restorations and the resin cement are clinically important for long-term use (10,11). The bonding process also influences microleakage between the tooth and adhesive cement which can cause bacterial invasion and seconder caries (12). Mechanical surface treatment methods, such as acid etching, air particle etching, and surface roughening with diamond rotary instruments, have been recommended to increase bond strength by providing micromechanical retention (13). Evaluation the effect of mechanical surface treatments on the adhesive surfaces are determined using imaging methods such as light microscopy, confocal microscopy, atomic force microscopy (AFM), scanning tunneling microscopy (STM), and scanning electron microscopy (SEM) (14).

Shear bond strength (SBS) and tensile bond strength are the two techniques used to evaluate bond strength (15). Lower bond strength is the outcome of SBS because it creates a considerably more severe stress concentration at the adhesive interface. It is recommended to employ tiny bonding areas to lessen this issue (16). Contrarily, in a study evaluating bonding strength between 3D resins and adhesive cements, it was reported that higher bonding strength values were obtained in the SBS compared to the tensile bond strength (17).

Previous research on the mechanical characteristics of 3D-printed permanent resins revealed that these materials’ mechanical attributes are similar to those of CAD/CAM blocks (4,18). But the evaluation of mechanical properties alone does not guarantee long-term intraoral use. Limited information about the bond strength of 3D-printed permanent resins is presented. This study aimed to evaluate the effect of surface treatment methods and 3D printer technology on the SBS between 3D-printed permanent resins and adhesive cement. The study’s null hypothesis is that surface treatment methods and 3D printer advancements won’t have an impact on the strength of the bond between adhesive resin and 3D-printed permanent resins.

## Material and Methods

-Preparation of Samples

In this study, samples were prepared from 3D-printed permanent restoration resins (Crowntec, Saremco Dental AG, Rebstein, Zwitzerland and Permanent Crown, Formlabs, MA, USA) using two different 3D printers: SLA (Formlabs 3B+, Formlabs, MA, USA) and DLP (Asiga MAX UV, Asiga, Sydney, Australia). The commercial names, manufacturers, composition, and lot number of 3D-printed permanent resins are listed in  Table 1.

G Power software (Heinrich-Heine-Universitat Düsseldorf, Germany) was used for power analysis. A total of 90 specimens (large effect size, α = 0.05, 1−β = 0.80) were analyzed and split into two study groups (SLA and DLP printer) with n = 45 each.

Samples with a rectangular prism form (12 x 8 x 2 mm3) were created using SLA or DLP 3D printer. For the constructed platform, the printing orientation was found to be 0 degrees, and each printed layer’s thickness was fixed at 50 µm.

Samples were created in the SLA group using Permanent Crown resin (Formlabs, MA, USA). Following the 3D printing procedure, the samples were washed using an isopropyl alcohol-containing automatic washing machine (FormWash, Formlabs, MA, USA) for three minutes. FormCure (Formlabs, MA, USA) was used for post-curing, and it was done for 40 minutes at 60°C.

As instructed by the manufacturer, samples were created in the DLP group using Crowntec resin (Saremco Dental AG, Switzerland). Following 3D printing, samples were immersed in 99% isopropyl alcohol for one minute, and an LED dual-mode light curing unit (Labolight DOU, GC, Japan) was used for 10 minutes of post-curing.

-Surface treatment 

The samples were embedded into acrylic resin blocks for the SBS test. The adhesive surfaces of samples were polished with P600 silicon carbide sandpaper under water cooling for one minute to obtain standardized surfaces. The samples were then ultrasonically cleaned in distilled water for five minutes in an ultrasonic cleaner (VGT-1740QTD, China). The SLA and DLP groups were divided into 3 subgroups (n:14) according to surface treatment methods:

Group 1: The samples were subjected to air abrasion for 10 seconds at 2.5 bar using 50 µm alumina oxide particles (Korox, Bego, Germany) at a 45-degree angle and a distance of 10 mm. Following a 5-second distilled water wash, the samples were allowed to air dry.

Group 2: 9.5% hydrofluoric acid (Porcelain Etchant, Bisco, USA) was applied for 60 s, the samples were then washed in distilled water for 10 s and air dried.

Group 3: Control group, no surface treatment was applied.

-Surface roughness

The surface features of the samples produced with the 3D printer, formed after different surface treatments, were examined with a confocal microscope (Smartproof 5, Zeiss, Germany). Initially, the surface roughness and topography of all samples were evaluated using the arithmetic mean roughness (Ra) and area-related mean arithmetic height (Sa) parameters. Analyzes were analyzed on images taken from a 0.5*0.5 mm area using a 20x.0.7 lens. Image processing and evaluation were performed with a surface metrology software (ConfoMap ST 9.3.10494; Zeiss, Germany).

-Bonding procedure

First, a primer (G-Multi Primer; GC, USA) was applied and air-dried. A transparent tube (diameter 2.38 mm and height 3 mm) was then placed in the middle of the adhesive surface, as specified in the ISO 29022:2013 standards (19). Adhesive cement (G-Cem ONE; GC, USA) was applied into using the stirring tip. After removing the excess cement, adhesive cement was polymerized with a curing light unit (DTE O-light, Woodpecker, Borkstrasse, Germany) for 20 s by placing the tip of the curing light unit on the upper surface of the tube. After the polymerization process, the tube was carefully removed by using a scalpel. All samples were stored in water (37˚C) for 24 h before SBS testing (20).

-SBS test

All samples were stored in water (37˚C) for 24 h before testing.20 Universal testing machine (H5KS, Tinius Olsen, Redhill, England) with 5kN of load cells (load measurement accuracy ± 0.5%) was used for the SBS test. The samples (n=14) were placed on the SBS tester holder. The cutting rod was applied to the 3D permanent resin-adhesive cylinder adherent interface at a speed of 0.5 mm/min until fracture occurred. The speed of the cutting blade was adjusted to 0.5 mm/min. By dividing the maximum load (N) by the adhesive surface area, bond strength was computed. Megapascals (MPa) were used to record the results.

-Microscopic analysis

Following the SBS test, an optical stereomicroscope with a 25x magnification (Leica MZ12, Houston, USA) was used to evaluate the different types of fractures on the adhesive surface. Adhesive fractures occurred between the sample and the luting composite; cohesive fractures occurred inside the luting composite resin; mixed cohesive fractures occurred within the 3D-printed resin (21). After that, the sample for each group was analyzed qualitatively (SEM pictures) at 5000 magnifications using scanning electron microscopy (ZEISS EVO 40, Carl Zeiss, Jena, Germany).

-Statistical analysis

The SBS data in the study were analyzed using the SPSS 22.0 (SPSS Inc., Chicago, IL, USA). The homogeneity of variance (α=0.05) was tested using the Kolmogorov-Smirnov test. To assess the impact of surface treatment techniques and 3D printing technology, a two-way analysis of variance (ANOVA) was conducted. The differences were compared using the post hoc Tukey test (*p*<0.05).

## Results

**Figure 1:**
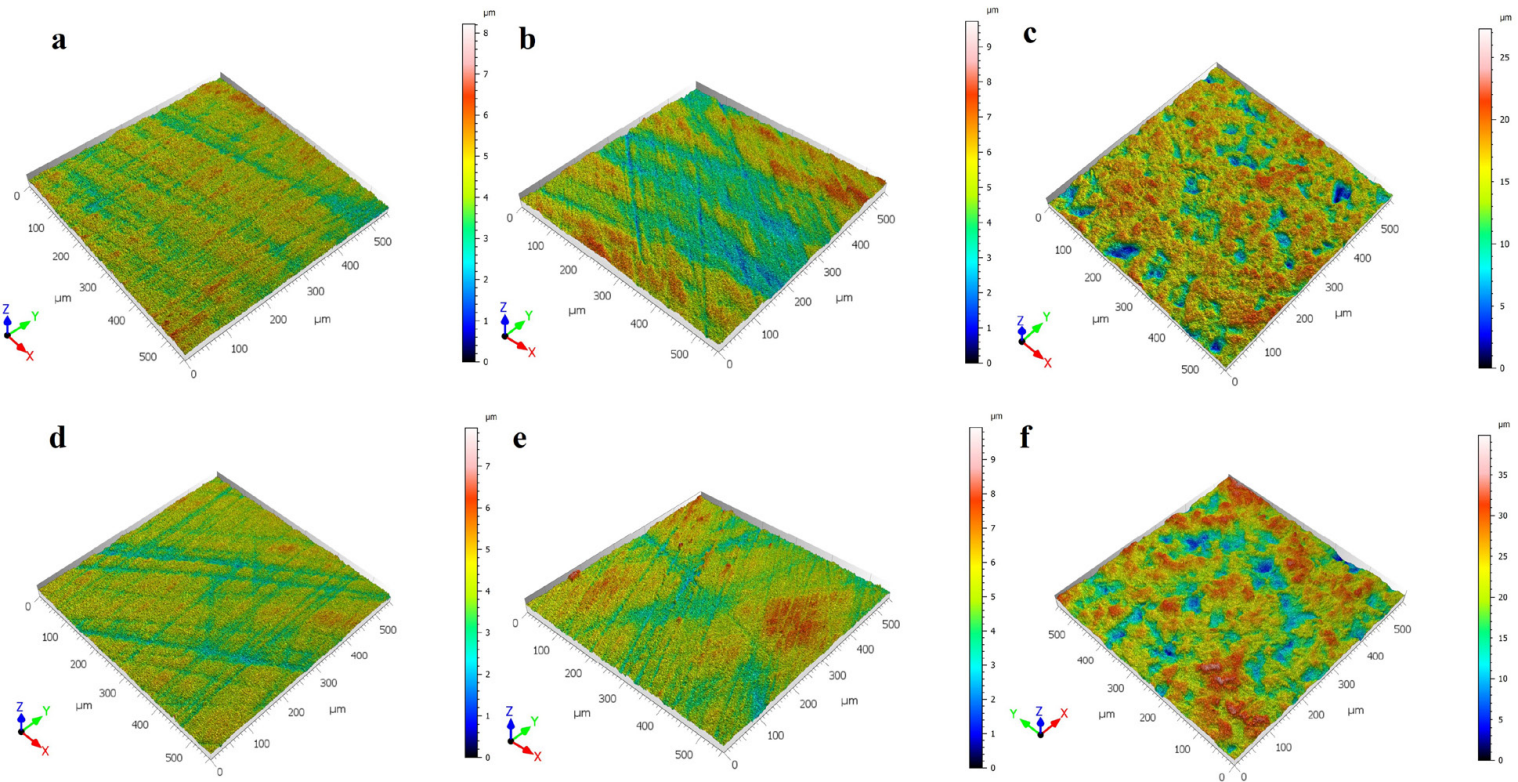
Investigation of the effect of different surface applications on 3D-printed permanent restoration resins by confocal microscope analysis. DLP 3D printer; a: sandblasting, b: hydrofluoric acid-etching, c: control (no treatment); SLA 3D printer d: sandblasting, e: hydrofluoric acid-etching, f: control (no treatment).

When the surface morphology of the 3D printer samples used in the study was examined with a confocal microscope; The samples with sandblasting applied to the surface showed higher surface roughness values than the samples with hydrofluoric acid applied (*p*<0.001), ( Table 2). Although hydrofluoric acid application increased the surface roughness, it was not statistically significant compared to the surface roughness value of untreated samples (*p*>0.05). When comparing the surface roughness values of the samples printed using the DLP and SLA printers, there was no statistically significant difference (*p* *p*>0.05). 3D surface topographies of samples with different surface preparations are shown in Figure 1. When confocal microscope images were examined, sandblasting caused intense topographical surface changes (Fig. 1a,d). Hydrofluoric acid-etching caused less microscopic modifications in the SLA and DLP sample (Fig. 1b,e). No microscopic changes were observed in control samples (Fig. 1c,f).

**Figure 2:**
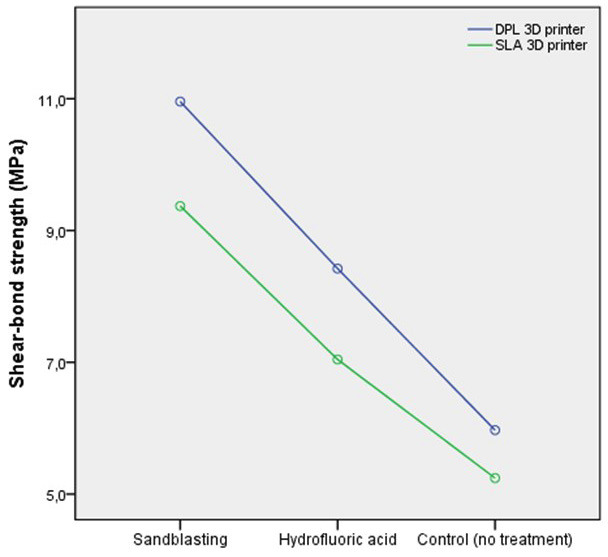
Examination of SBS values according to 3D printer type. Blue: DLP 3D printer, Green: SLA 3D printer.

Interactions between 3D printer technology and surface treatment methods for SBS were significant (*p*<0.001). 3D printer technology and surface treatment method self-adhesive resin cement also affected SBS values (*p*<0.001). The results of descriptive statistics (mean ±standard deviation) of SBS values are shown in  Table 3. Higher SBS values were noted in the DLP group than in the SLA group (*p*<0.001). Higher SBS values were noted in the sandblasting group (DLP: 11.6±1.3, SLA: 9.6±1.1) than in hydrofluoric acid (DLP: 9.9±0.8, SLA: 7.4±1.2) and control groups (DLP: 8.4±1.1, SLA: 6.1±1.3), (*p* < 0.001), (Fig. 2).

**Figure 3:**
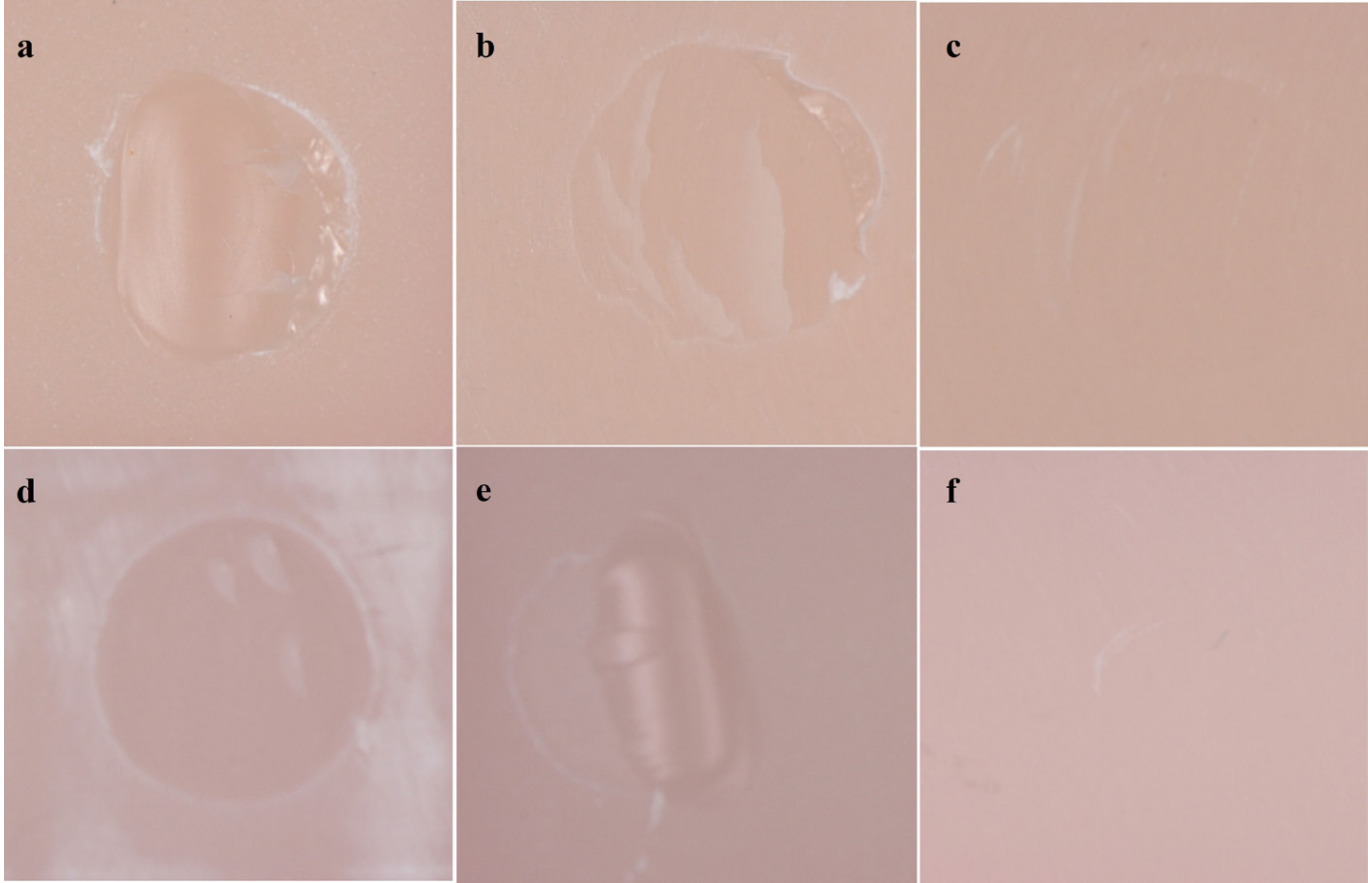
Fracture types on the surface of 3D-printed permanent restoration resins specimens subjected to SBS testing. DLP 3D printer; a: sandblasting, b: hydrofluoric acid-etching, c: control (no treatment); SLA 3D printer; d: sandblasting, e: hydrofluoric acid-etching, f: control (no treatment).

While cohesive fractures were mostly observed in sandblasted DLP (Fig. 3a) and SLA (Fig. 3d) samples, mix fractures were observed in hydrophilic acid-etching groups (Fig. 3b,e), and adhesive fractures were observed in the control groups (Table 4, Fig. 3c,f).

**Figure 4:**
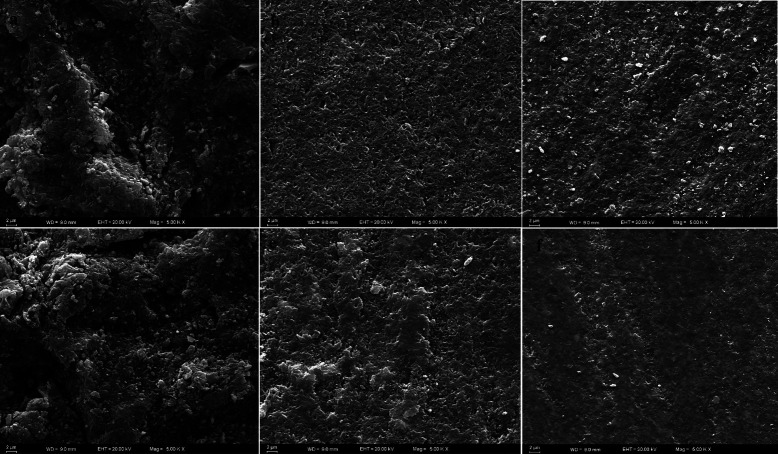
Investigation of the effect of different surface applications on 3D-printed permanent restoration resins by SEM analysis. DLP 3D printer; a: sandblasting, b: hydrofluoric acid-etching, c: control (no treatment); SLA 3D printer d: sandblasting, e: hydrofluoric acid-etching, f: control (no treatment).

SEM images were given in Figure 3. When SEM images were examined, sandblasting caused intense topographical surface changes (Fig. 4a,d). Hydrofluoric acid-etching caused less microscopic modifications in the DLP and SLA sample (Fig. 4b,e). No microscopic changes were determined in control samples (Fig. 4c,f).

## Discussion

The effect of 3D printing technology and surface treatment methods on SBS between 3D permanent resins and adhesive cement was evaluated. The results of this study showed that 3D printing technology and surface treatment methods affected SBS between 3D permanent resins and adhesive cement. So, the null hypothesis of this study was rejected.

Surface treatment methods are recommended to achieve durable bond strength between indirect restorations and adhesive cements by increasing adhesive area, surface energy, and wettability of indirect restorative material (22). Sandblasting causes irregular microgrooves on the surface of restorative material, while hydrofluoric acid dissolves the filler particles in the structure and creates a porous surface (23). Sandblasting provides a rougher surface which increases micromechanical retention between the resin cement and material interface (24). Although there are studies stating that sandblasting should be preferred for surface treatment of indirect restorations to achieve a dependable bonding (25,26) there are also studies stating that hydrofluoric acid etching is an effective surface treatment method for resin-based CAD-CAM restorations and was recommended instead of sandblasting (27,28) In a previous study, higher surface roughness values were observed by sandblasting (ranging from 1.32 to 1.98 μm) than by hydrofluoric acid etching (ranging from 0.12 to 0.46 μm) in the resin-based CAD/CAM blocks (29).

In our study, sandblasted samples showed higher surface roughness values than the literature. However, surface roughness values similar to the values in the literature were obtained in the samples treated with hydrofluoric acid. Sandblasting application created more rough surfaces on the surface of the samples produced with the 3D printer. Therefore, higher SBS values were achieved by sandblasting of the 3D-printed permanent resins than by hydrofluoric acid-etching. In addition, in the SEM analysis, it was observed more topographical changes on the sandblasted samples.

Although sandblasting and hydrofluoric acid-etching on the material surface promote micromechanical bonding, the primer application is recommended to increase bonding. For this purpose, primers with silane, especially 10-methacryloyloxydecyl dihydrogen phosphate (10 MDP), are applied to the adhesive surface after surface treatment. Primers increase the wettability of the material surface and penetrate deep into the micro-cracks created by surface treatment (30). Silane consists of organofunctional molecules with two functional groups that can react with ceramic and resin materials. While hydrolysable functional groups react with silicon-linked hydroxyl groups (Si-OH) in ceramics, organic functional groups react with organic monomers in adhesives and composite resins (31). In previous studies the use of “universal primers” has been shown to increase the bond strength of adhesive cements to both the tooth substrate and the restorative materials (32,33). In this study, since 3D-printed permanent resins contain silica particles, a universal primer was applied to the adhesive surfaces prior to adhesive cement application.

3D-printed permanent resins have the content of a relatively low filler (< 50% by weight) to ensure printing accuracy. However, resin-based CAD/CAM materials generally contain higher fillers (>70% by weight). The filler ratio and size of resin-based CAD/CAM materials influence SBS (24,34). Miyazaki at al. (35) reported that bond strength increased with increasing filler content. In another study, it was determined that the lower filler ratio and degradation of the matrix of the 3D printed resins (temp print and GR-17) adversely affected SBS values (36). In this study, it was observed that cohesive fractures occurred in 100% of the fractures of sandblasted samples and 50% of fractures of the hydrofluoric acid-etched samples. The low filler ratio of 3D-printed permanent resins could explain the cohesive fractures of 3D permanent resins. In this study, artificial aging procedures were not performed. Future studies are needed to evaluate the damaging effects of surface treatment methods on matrix degradation and bond strength in long-term use.

The principle of vat polymerization is to use a certain wavelength of light to polymerize the liquid resin (37). The resolution of the x, y, and z axes, which is related to the features of the 3D printer technology, determines the precision of 3D printing. The smallest feature size that a 3D printer can reproduce horizontally is known as the XY axis. The diameter of the laser point determines the resolution of SLA printers, whereas the pixel size of the projector or LCD screen determines the resolution of DLP printers. 3D printing process and resin may have an impact on the Z-axis resolution, which measures the printing layer thickness (38). After 3D printing process, physical properties such as mechanical strength are insufficient, post-curing process is required to increase the polymerization rate. However, post-curing process does not guarantee a homogeneous polymerization which might influence mechanical strength of 3D resins. Additionally, post-curing time also influences long-term mechanical strength (39). Besides, post-curing increases the degree of conversion on the resin surface. However, surface treatment procedures are assumed to positively affect the bonding between the 3D resin matrix and the luting composite by exposing unreacted double bonds under the 3D resin surface (39,40). In this study, higher SBS values between 3D resin and luting composite were noted in DLP samples than in SLA samples. The differences in post-curing process may cause different degree of conversion values on resin surfaces. After the surface treatment applications, unreacted double bonds present under the surface at different rates may have been exposed and may have caused differences in SBS values. The effect of post-curing on the degree of conversion of resin layers should be evaluated in future studies.

This study had certain limitations because it only examined a small number of samples *in vitro*. Conditions within the mouth, like the presence of saliva and its components, different chewing forces in different directions and magnitudes, and temperature changes, were not considered. The literature will benefit from clinical follow-up studies of permanent restorations made with 3D printers in the future.

## Conclusions

Within this *in vitro* study’s limitations:

Sandblasting created greater surface roughness on 3D printed samples. The sandblasting groups obtained higher SBS values. In the sandblasting groups, cohesive fractures were seen, while in the hydrofluoric acid-etching groups, mix fractures were seen. Cohesive fractures of 3D-printed permanent resins might be caused by the low filler ratio of the materials. Higher SBS values were achieved in DLP 3D printer samples than in SLA 3D printer samples.

## Figures and Tables

**Table 1:** 3D printer resins used in the study.

Materials	Composition by weight		Lot Number
	Filler (wt)	Polymer	
Crowntec (Saremco Dental AG, Zwitserland)	Inorganic fillers (particle size 0.7 μm) are 30-50% by mass.	4,4'isopropylidiphenol, ethoxylated and 2-methylprop-2enoic acid, silanized dental glass, pyrogenic silica, initiators	E175
Permanent Crown (Formlabs, USA)	Inorganic fillers (particle size 0.7μm) are 30– 50% by mass	4,4'-isopropylidiphenol, ethoxylated and 2-methylprop-2enoic acid, silanized dental glass, methyl benzoylformate, diphenyl (2,4,6-trimethyl benzoyl) phosphine oxide	600394
Self-adhesive resin cement	-	Paste A: Fluoroaluminosilicate glass, methacrylic acid ester, initiator Paste B: Silica filler, methacrylic acid ester, phosphoric methacrylate monomer,initiator	2204131
Primer	-	Vinyl silane, phosphoric methacrylate monomer, thiophosphoric ester monomer, methacrylic acid ester, ethyl alchol	2203181

**Table 2:** Surface roughness values (Ra and Sa) of 3D-printed permanent resins according to different surface treatments.

Materials/Surface treatments	Surface arithmetic mean roughness (Ra, µm)		Surface area-related mean arithmetic height (Sa, µm)	
	DLP 3D printer	SLA 3D printer	DLP 3D printer	SLA 3D printer
Sandblasting	2.3±0.4^a,A^	2.5±0.2^a,B^	3.3±0.6^a,A^	4.1±0.1^b,B^
Hydrofluoric acid	0.5±0.1^b,A^	0.5±0.1^b,A^	0.7±0.1^b,A^	0.6±0.1^b,A^
Control (no treatment)	0.4±0.1^b,A^	0.4±0.1^b,A^	0.6±0.1^b,A^	0.6±0.1^b,A^

*The limit of significance among surface treatments (a–b) and between (A–B) 3D printer. *P*< 0.05.

**Table 3:** SBS values (Mean±SD) of 3D-printed permanent resins according to different surface treatments.

Materials/Surface treatments	DLP 3D printer	SLA 3D printer
Sandblasting	11.6±1.4^a,A^	9.6±1.1^a,B^
Hydrofluoric acid	9.9±0.8^b,A^	7.4±1.2^b,B^
Control (no treatment)	8.4±1.2^c,A^	6.1±1.3^c,B^

*The limit of significance among surface treatments (a–c) and between (A–B) 3D printer. *P*< 0.05.

**Table 4:** Investigation of fracture types of 3D-printed permanent resins according to different surface treatments.

Materials	Surface treatments	Adhesive	Mix	Cohesive
DLP 3D printer	Sandblasting	0	0	14
	Hydrofluoric acid	2	8	4
	Control (no treatment)	14	0	0
SLA 3D printer	Sandblasting	0	4	10
	Hydrofluoric acid	10	4	0
	Control (no treatment)	14	0	0

## Data Availability

The datasets used and/or analyzed during the current study are available from the corresponding author.
